# Developing an Automatic System for Classifying Chatter About Health Services on Twitter: Case Study for Medicaid

**DOI:** 10.2196/26616

**Published:** 2021-05-03

**Authors:** Yuan-Chi Yang, Mohammed Ali Al-Garadi, Whitney Bremer, Jane M Zhu, David Grande, Abeed Sarker

**Affiliations:** 1 Department of Biomedical Informatics School of Medicine Emory University Atlanta, GA United States; 2 Division of General Internal Medicine and Geriatrics Oregon Health & Science University Portland, OR United States; 3 Division of General Internal Medicine Perelman School of Medicine University of Pennsylvania Philadelphia, PA United States; 4 Department of Biomedical Engineering Georgia Institute of Technology and Emory University Atlanta, GA United States

**Keywords:** natural language processing, machine learning, Twitter, infodemiology, infoveillance, Twitter, social media, Medicaid, consumer feedback

## Abstract

**Background:**

The wide adoption of social media in daily life renders it a rich and effective resource for conducting near real-time assessments of consumers’ perceptions of health services. However, its use in these assessments can be challenging because of the vast amount of data and the diversity of content in social media chatter.

**Objective:**

This study aims to develop and evaluate an automatic system involving natural language processing and machine learning to automatically characterize user-posted Twitter data about health services using Medicaid, the single largest source of health coverage in the United States, as an example.

**Methods:**

We collected data from Twitter in two ways: via the public streaming application programming interface using Medicaid-related keywords (Corpus 1) and by using the website’s search option for tweets mentioning agency-specific handles (Corpus 2). We manually labeled a sample of tweets in 5 predetermined categories or *other* and artificially increased the number of training posts from specific low-frequency categories. Using the manually labeled data, we trained and evaluated several supervised learning algorithms, including support vector machine, random forest (RF), naïve Bayes, shallow neural network (NN), k-nearest neighbor, bidirectional long short-term memory, and bidirectional encoder representations from transformers (BERT). We then applied the best-performing classifier to the collected tweets for postclassification analyses to assess the utility of our methods.

**Results:**

We manually annotated 11,379 tweets (Corpus 1: 9179; Corpus 2: 2200) and used 7930 (69.7%) for training, 1449 (12.7%) for validation, and 2000 (17.6%) for testing. A classifier based on BERT obtained the highest accuracies (81.7%, Corpus 1; 80.7%, Corpus 2) and F_1_ scores on consumer feedback (0.58, Corpus 1; 0.90, Corpus 2), outperforming the second best classifiers in terms of accuracy (74.6%, RF on Corpus 1; 69.4%, RF on Corpus 2) and F_1_ score on consumer feedback (0.44, NN on Corpus 1; 0.82, RF on Corpus 2). Postclassification analyses revealed differing intercorpora distributions of tweet categories, with political (400778/628411, 63.78%) and consumer feedback (15073/27337, 55.14%) tweets being the most frequent for Corpus 1 and Corpus 2, respectively.

**Conclusions:**

The broad and variable content of Medicaid-related tweets necessitates automatic categorization to identify topic-relevant posts. Our proposed system presents a feasible solution for automatic categorization and can be deployed and generalized for health service programs other than Medicaid. Annotated data and methods are available for future studies.

## Introduction

Consumers’ perspectives and feedback are crucial for improving products or services. Over the last two decades, widespread adoption and use of the internet has led to its use as a major platform for collecting targeted consumer feedback. Businesses often allow consumers to rate specific products and services and provide detailed comments or reviews, and this has become a key feature of e-commerce platforms. For example, consumer-generated reviews and ratings of products play an important role in the differentiation on Amazon’s e-commerce site, which currently has a global presence [1,2]. There are also companies, such as Yelp, that focus specifically on crowdsourcing consumer feedback [3-6]. Similarly, as social media has become the primary platform of communication for many people, many companies have started maintaining and communicating via social media accounts, often enabling direct communications, both private and public, with consumers. Not only do consumers provide comments or seek assistance through these social media accounts, they also often engage in discussions about products or services within their own social networks. Consequently, such consumer-generated chatter is often used to assess perceptions about specific topics, which may range from products or services to social programs, legislation, and politics.

Social media is a rich resource for obtaining perspectives on public health, as it enables the collection of large amounts of data directly and in real time. It is commonly used for sentiment analysis—a field of study that analyzes opinions, sentiments, attitudes, and emotions from written language. Sentiment analysis research involving social media data has covered a wide range of topics, events, individuals, issues, services, products, and organizations [7,8]. However, the use of social media has not been limited to sentiment analysis in open domains. In recent years, research within the broader medical domain has embraced social media, and it is currently being used for conducting real-time public health surveillance, including for topics such as influenza surveillance, pharmacovigilance, and toxicovigilance [9-11]. Meanwhile, similar to corporate businesses in the United States, health service providers such as local health departments and hospitals have also started adopting social media specifically as a consumer-facing communication channel [12,13]. Prior studies in this area have investigated how social media data linked to such health services accounts reflect the consumers’ perspectives about them. The simplest studies have focused on using structured or numeric information, such as likes or ratings, associated with the accounts belonging to hospitals or nursing homes, and these metrics have been compared with traditional quality reports and ratings [14-16]. Building on the advances in open-domain natural language processing (NLP), some studies within the broader health domain have attempted to use unstructured data, including postings related to patients’ experiences about hospitals, to infer consumer sentiments [17,18] or extract topics that summarize content [19].

Extracting knowledge from social media data is notoriously difficult for NLP methods because of factors such as the presence of misspellings, colloquial expressions, lack of context, and noise. These problems are exacerbated for health-related data because of the complexities of domain-specific terminologies, the lack of expert knowledge among common social media users, and the uniqueness of health-related topics. Consequently, there is considerably less research using the free-text data on social media for health-related tasks. Past studies closely related to ours have focused on analyzing sentiments toward attributes of health insurance plans [20] and social media users’ responses to public announcements about health policies [21]. However, to the best of our knowledge, there has been no near real-time automatic system that provides comprehensive data collection and analysis on social media chatter about health services and insurance coverage provided by large public insurers such as Medicaid and Medicare.

Nevertheless, such a system is essential for analyzing the public’s perspective toward public insurers and their governance and policies from social media. For example, the customers using Medicaid might provide their feedback based on their experiences or even engage in discussion of their experiences, which they might not have a chance or even willing to reveal to the Medicaid providers’ customer service representatives. Analyzing such chatter could provide researchers and policy makers with information complementary to traditional customer feedback channels and possibly help improve the services and related policies. However, chatter associated with an entity such as Medicaid contains discussions about politics and legislation; academic research, statistics, and factual information; consumer feedback; and so on. Chatter related to politics can be very different in terms of content, compared with chatter related to consumer feedback. Thus, properly categorizing these tweets based on content is crucial for providing accurate information. Furthermore, sentiment may also have different meanings for these 2 broad categories of chatter—negative sentiment in political chatter may represent a user’s emotions associated with a political decision about the health service (eg, changes in policies related to insurance coverage or covered benefits within Medicare or Medicaid) rather than the service itself.

Therefore, there is a need to identify and categorize the content of the chatter before it can be used for targeted analyses. A good categorization scheme can not only help in bringing forth good analysis but can also help to avoid contaminating the chatter with irrelevant content. To achieve this and effectively use social media big data, automatic classification and analysis systems based on machine learning methods are required. This, together with the promise of social media data and the lack of past research in this specialized area, served as the primary motivation for this study. We chose Medicaid as our target health service because it is the single largest public insurance program in the country [22] and contains large volumes of related chatter on social media.

**Figure 1:**
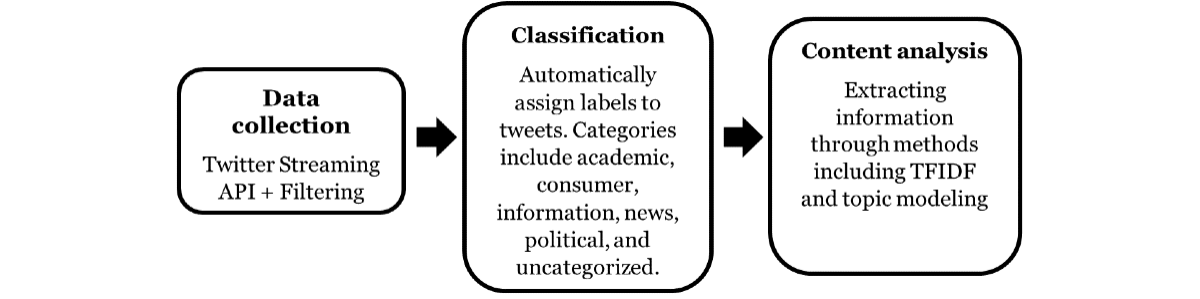
Workflow of the natural language processing system for automatic data collection, classification, and content analysis of the Medicaid chatter on Twitter. API: application programming interface; TFIDF: term frequency-inverse document frequency.

The specific objectives of this paper are as follows:

- To assess if a social media platform, specifically Twitter, contains sufficient volumes of chatter about health services so that it can be used to conduct large-scale analyses, using Medicaid as our target service
- To develop and discuss a data-centric system involving NLP and machine learning to automatically collect, categorize, and analyze Twitter chatter associated with Medicaid, as shown in Figure 1
- To describe the manual annotation of a Twitter-Medicaid data set and its composition
- To describe supervised classification strategies for automatically classifying Medicaid-related tweets into broad categories and evaluating the performances of several machine learning models, with particular emphasis on tweets that potentially represent consumer feedback
- To conduct postclassification content analyses to verify the potential utility of our data-centric system

The main contributions of this paper are as follows:

- We present the methods and results of collecting Medicaid-related Twitter data, analyzing a sample of the data manually, and developing an annotation guideline suitable for preparing a large data set for training classification algorithms.
- We present details of automatic supervised classification experiments, including methods, results, and evaluations, and provide suggestions on how to further improve the performance.
- We discuss the postclassification analyses of the collected data, including data distribution and content analyses.
- We make the NLP and machine learning scripts in this study publicly available, along with the labeled training data set and a larger set of unlabeled Medicaid-related data.

## Methods

### Data Collection

To develop our models for analyses of Twitter data related to Medicaid, we collected 2 sets of publicly available data from the network, which we labeled Corpus 1 and Corpus 2. Corpus 1 contains tweets mentioning the term “Medicaid,” or Medicaid agency (MA) and managed care organization (MCO, an organization that provides Medicaid-related health services under contracts from the agency) names that are branded and thus easily distinguishable on Twitter (eg, *Medi-cal*: California’s Medicaid program, and *TennCare*, Tennessee’s Medicaid program). These tweets were collected via Twitter’s public streaming application programming interface from May 1, 2018, to October 31, 2019, and were limited to only English tweets. It has been reported that misspellings appear frequently on social media platforms [23], particularly Twitter; hence, we used an automatic spelling variant generator to generate common misspellings for “medicaid” and used them to capture tweets referring “medicaid” as one of the misspellings [24]. This can increase the retrieval rate and increase the volume of streaming data. The full list of keywords, including misspellings, is shown in Table S1 in Multimedia Appendix 1. We then identified and removed tweets whose contents were not directly related to Medicaid and tweets with repeated or duplicated contents (eg, fundraising or political campaigns). To focus on tweets expressing personal opinions, we removed retweets, which were deemed as duplicates of the original tweets. The final data set consisted of 628,411 tweets for Corpus 1.

Although most of the chatter regarding Medicaid posted by consumers only included the term “medicaid” (or its variants), some directly tagged or mentioned relevant Twitter handles associated with MAs or the MCOs (eg, “@organization_name”). Corpus 2 is composed of such tweets, and the MA and MCO Twitter handles were identified in a previous study [25]. The full list of the handles used in data collection is presented in Table S2 in Multimedia Appendix 1. These tweets were retrieved by targeted searching (eg, “to:organization_name”) on Twitter. These tweets were posted between December 12, 2008, and the time of search (January 9, 2020). We filtered the tweets using the same approaches used for Corpus 1. Overall, there were 27,337 tweets in the corpus. Additional notes regarding our data are provided in Multimedia Appendix 1.

### Tweet Contents and Manual Annotations

To better understand the contents of the tweets posted by users and to develop methods to automatically characterize the posts, we first performed manual inspections of the contents of the posts and identified commonly occurring themes. We used the grounded theory approach to conduct a thorough analysis [26]. We analyzed a random sample of tweets to identify recurring topics and then grouped the topics into broader categories and themes. The analysis was conducted by multiple authors of this paper, and the topics discovered initially were discussed. The discovered topics were either merged into broader themes (eg, combining *information* and *outreach*), discarded from our consideration (eg, for topics that were observed rarely or only once), or split into multiple themes (eg, splitting of *information* tweets into *academic*, *information*/*outreach*, and *news*). Following iterative discussions and finalization by the domain expert authors of this paper (JMZ and DG), we classified tweet contents into 5 broad categories: (1) academic, (2) consumer feedback, (3) information/outreach, (4) news, and (5) political opinion/advocacy. Tweets that could not be categorized as any of these were labeled as *other*. The descriptions of these classes are as follows:

- *Academic *(*academic*)—tweets related to research on Medicaid. These included tweets by persons or organizations with academic affiliations or think tanks that expressed the perspective from the affiliated organizations or any tweet relating to education, scholarship, and thought, including (links to) journal publications and reports.
- *Consumer feedback* (*consumer*): These included tweets related to consumers’ experiences or questions related to Medicaid services, coverage, benefits, or health issues. The tweets were typically from Medicaid consumers or family members of consumers and also included discussions with others.
- *Information/outreach* (*information*): These included tweets directed at consumers and beneficiaries of Medicaid to convey information including agency services, programs, events, enrollment, eligibility criteria, etc. Tweets containing information about general health or public health reminders were also included.
- *News* (*news*): These included news and announcements, including any tweets from a news agency or organization. Tweets that explicitly expressed political opinions and tweets from Medicaid agencies or plans were excluded.
- *Political opinion/advocacy* (*political*): These included comments, personal opinions, and feedback about politics related to Medicaid.
- *Other* (*other*): These included tweets that were not relevant, typically the noise that is not captured by the initial screening.

Following the establishment of the desired categories and the development of annotation guidelines by JMZ, 2 trained annotators performed the first round of annotations (for the data in Corpus 1) in multiple iterations, developed annotation guidelines, and resolved ambiguities via discussions. Following the completion of this round of annotations, the annotation disagreements were resolved by AS and WH. We found the class distribution to be very imbalanced, with most of the tweets annotated as *news*, *political*, and *other*, whereas only a small portion were in *academic*, *consumer*, and *information* categories (Table 1). Examples of each category are provided in Table S3 in Multimedia Appendix 1. To understand how this imbalanced distribution affected the classifier performances on the smaller classes, particularly the consumer class, we performed preliminary automatic classification experiments using 3 classifiers: naïve Bayes (NB), support vector machine (SVM), and random forest (RF). We split the data into training (5795/7244, 80%) and validation (1449/7244, 20%) sets and found the best performance on consumer feedback to be low for all the classifiers, with the best F_1_ score=0.3 (SVM). Tweets belonging to the consumer feedback class were of particular importance to our overarching project objectives, so we devised 2 strategies for improving performance for this class: the first involved additional annotations of targeted tweets from the same data set and the second focused on collecting an additional data set (Corpus 2, as described earlier).

**Table 1:** Distribution (counts and percentages) of annotated data in the first round of annotations (rows 2 and 3) and the final data sets (Corpus 1 for rows 4 and 6; Corpus 2 for rows 5 and 7).

Data set	Academic, n (%)	Consumer, n (%)	Information, n (%)	News, n (%)	Political, n (%)	Other, n (%)	Total, n (%)
Training set (first round)	61 (1.05)	158 (2.73)	198 (3.42)	1288 (22.23)	3613 (62.34)	477 (8.23)	5795 (100)
Validation set (first round)	35 (2.42)	37 (2.55)	49 (3.38)	317 (21.88)	897 (61.90)	114 (7.86)	1449 (100)
Training set (Corpus 1)	83 (1.23)	355 (5.27)	429 (6.37)	1299 (19.30)	3710 (55.13)	854 (12.69)	6730 (100)
Training set (Corpus 2)	9 (0.75)	709 (59.08)	94 (7.83)	40 (3.33)	10 (0.83)	338 (28.17)	1200 (100)
Test set (Corpus 1)	20 (2)	46 (4.60)	49 (4.90)	199 (19.90)	603 (60.30)	83 (8.30)	1000 (100)
Test set (Corpus 2)	6 (0.60)	579 (57.90)	80 (8.00)	21 (2.10)	6 (0.60)	308 (30.80)	1000 (100)
Total	153 (1.34)	1726 (15.17)	701 (6.16)	1876 (16.49)	5226 (45.93)	1697 (14.91)	11,379 (100)

For the first strategy, we conducted another round of annotation of tweets from Corpus 1 to increase the number of tweets for the consumer class. Owing to the very low number of consumer class tweets in the original data set, we realized that it would not be feasible to annotate sufficient numbers of these tweets by drawing random samples because of budgetary and other constraints. Therefore, rather than randomly drawing tweets for the next round of annotations, which would again lead to finding a small number of tweets belonging to the consumer feedback category, we attempted to artificially increase the number of tweets for this category. We achieved this by running our above-described weak classifier on a larger set of unlabeled tweets and only picking tweets classified as consumer feedback by the SVM classifier. This significantly increased the number of consumer feedback tweets in the data to be annotated. The new set of annotated data was then added to the training set, and the data distribution is presented in Table 1.

**Figure 2:**
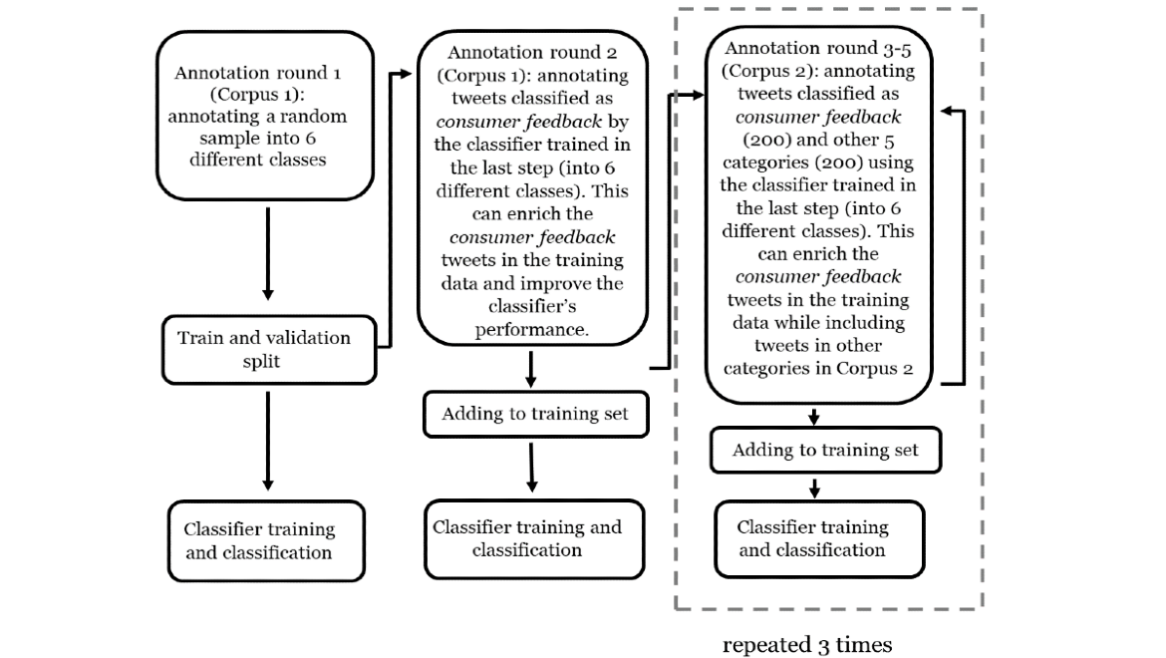
Flowchart for the entire annotation process (for the training and validation set) involving multiple rounds.

We followed the same annotation strategy for Corpus 2 (ie, annotating tweets classified as consumer feedback by the classifier trained on the previously annotated data), but this time, we also annotated equal amount of nonconsumer tweets. This is because Corpus 2 is rich in consumer feedback tweets, and we would also like to include tweets in other categories to improve performance. An outline of the overall annotation process is presented in Figure 2. Although we tried to decrease the class imbalance in the training sets of the 2 corpora, to ensure that our evaluations represented the classifier performances on real-world distributions of the data, we did not artificially balance the validation set. We also annotated the test set randomly generated from the 2 corpora, 1000 tweets each, so they would reflect the data composition of the original corpora, allowing us to evaluate how the classifier would perform when deployed for streaming data.

### Classification

We experimented with 5 traditional classification algorithms, including Gaussian NB [27,28], SVM [29,30], RF [31], k-nearest neighbor (KNN) [28], and shallow neural network (NN), and 2 advanced classification algorithms, bidirectional long short-term memory (BLSTM) [32,33] and bidirectional encoder representations from transformers (BERT) [34,35]. Although the origin and distributions of tweets in the 2 corpora were different, we decided to combine them as our previous research suggests that multicorpus training, or distant supervision, leads to performance improvements for social media text classification [36]. The feature extraction and classification training for traditional classifiers was done using the “Scikit-learn” package in Python [37], the BLSTM classification was implemented using package “Keras” in Python [38], and the BERT classification approach was implemented using package “simpletransformers,” which is based on the package “transformers” [39]. The performance on the validation set and the test set from Corpus 1 and Corpus 2 are shown in Table 2.

**Table 2:** Classification performances of the classifiers on the test sets of Corpus 1 and Corpus 2.

Data set and classification algorithm		F_1_ score (0.XX)^a^							Percentage accuracy (95% CI)
		Academic	Consumer (95% CI)	Information	News	Political	Other		
**Validation set**									
	NB^b^	11	17 (11-24)	24	55	70	34	55.0 (52.4-57.6)	
	SVM^c^	0	*53 (38-66)* ^d^	26	70	87	43	77.4 (75.2-79.5)	
	RF^e^	5	43 (26-58)	27	74	87	48	78.7 (76.6-80.7)	
	KNN^f^	5	24 (12-37)	11	55	65	26	51.4 (48.9-54.0)	
	NN^g^	31	34 (21-46)	32	72	86	46	75.2 (72.9-77.4)	
	BLSTM^h^	27	38 (25-51)	42	74	88	53	78.9 (76.8-81.0)	
	BERT^i^	54	*61 (48-72)*	64	82	*92*	67	*85.2 (83.3-87.0)*	
**Test set (Corpus 1)**									
	NB	12	23 (16-31)	20	53	71	21	53.5 (50.4-56.6)	
	SVM	0	38 (24-51)	14	71	83	19	73.0 (70.2-75.7)	
	RF	0	24 (10-37)	21	75	84	24	74.6 (71.9-77.2)	
	KNN	0	20 (9-32)	15	47	66	26	49.0 (45.9-52.1)	
	NN	25	*44 (31-56)*	33	70	84	32	71.8 (69.0-74.6)	
	BLSTM	22	33 (19-45)	20	71	84	30	73.1 (70.4-75.8)	
	BERT	72	*58 (45-70)*	58	80	*89*	51	*81.7 (79.3-84.0)*	
**Test set (Corpus 2)**									
	NB	0	72 (69-75)	30	11	3	21	47.3 (44.2-50.3)	
	SVM	0	76 (73-78)	2	21	7	18	56.4 (53.3-59.4)	
	RF	0	*82 (80-84)*	7	16	11	66	69.4 (66.6-72.3)	
	KNN	0	38 (33-42)	0	7	0	50	42.2 (39.1-45.3)	
	NN	0	79 (76-82)	40	24	5	66	66.0 (63.0-69.0)	
	BLSTM	0	81 (79-84)	34	21	4	55	67.3 (64.4-70.2)	
	BERT	50	*90 (89-92)*	49	37	21	79	*80.7 (78.2-83.1)*	

^a^The number represents the first two decimal points. For example, the F1 score for SVM on Consumer is 0.53 with 95% CI 0.38-0.66.

^b^NB: naïve Bayes.

^c^SVM: support vector machine.

^d^The best scores are highlighted in italics.

^e^RF: random forest.

^f^KNN: k-nearest neighbor.

^g^NN: shallow neural network.

^h^BLSTM: bidirectional long short-term memory.

^i^BERT: bidirectional encoder representations from transformers.

The tweets were preprocessed by lowercasing and anonymizing the URLs and user names. For the traditional classifiers, the non-English characters were further removed (keeping underline), and each word was stemmed by the Porter stemmer. The features were the unnormalized counts of the 3000 most frequent n-grams (contiguous sequences of words with n ranging from 1 to 3, with 1380 unigrams, 1296 bigrams, and 324 trigrams). We also introduced a “word cluster” feature, which are clusters or generalized representations of semantically similar words or phrases learned from Twitter chatter [10,40]. The word clusters were represented as bag-of-word vectors, and the feature space consisted of 972 word clusters. We used the Twitter word clusters, “50mpaths2,” provided by Owoputi et al [41]. For the advanced classifiers, each word or character sequence was replaced with a dense vector, and the vectors were then fed into the relevant algorithms for training.

We performed hyperparameter tuning using the validation set to improve the classification task on the imbalanced data set. Specifically, we focused on improving the F_1_ score for consumer feedback. For traditional classifiers, we optimized the number of nearest neighbors for KNN, the number of estimators (trees) for RF, and the c parameter and weights for SVM. We also experimented with oversampling using the synthetic minority oversampling technique, but the performance was not improved (provided in Table S4 in Multimedia Appendix 1). The optimal hyperparameters for the traditional classifiers are listed in Table S5 in Multimedia Appendix 1. We used the Twitter GloVe word embeddings for the BLSTM [42] classifier, where each word was converted to a 200-dimensional vector. BLSTM was then trained with 40 epochs and dropout regularization, and the best model was selected based on the accuracy of the validation data. We chose RoBERTa-large for the BERT algorithms [35], trained with 3 epochs. The technical details are provided in Table S5 in Multimedia Appendix 1.

### Postclassification Analyses

To assess the utility of our classification approaches and gain an understanding of the data, we used the best-performing classifier (the classifier based on BERT) to label all collected unlabeled data and compute the data distribution. We then performed content analysis using the term frequency-inverse document frequency (TFIDF) method [43], focusing on the tweets in Corpus 1 that contained the term “medicaid” and its misspellings, and using the latent Dirichlet allocation (LDA) for topic modeling [44], focusing on consumer feedback tweets. Our first intent was to qualitatively assess whether the classifier was capable of distinguishing tweets based on contents that were manually verifiable. Second, we wanted to obtain a basic understanding of the content of each category by identifying the top rated TFIDF words. The TFIDF method adjusts the term frequencies with inverse document frequency so that the high-frequency terms unique in one category would rank higher than the high-frequency words that are common across the categories. This helps identify important terms unique to the target category. Our third objective was to summarize consumer feedback chatter using LDA topic modeling, going beyond the TFIDF method. For all content analyses, the text was first preprocessed by lowercasing and removing URLs, user names, non-English characters (keeping underline and hyphen), stopwords, and any word with less than 4 characters. For LDA topic modeling, we experimented with different hyperparameters (number of topics=5, 10, 20, 50, and 100) and selected the model with the highest coherence score.

## Results

### Annotation and Class Distributions in Test Sets

We annotated a total of 9179 tweets from Corpus 1 and 2200 tweets from Corpus 2. We obtained substantial interannotator agreement (Cohen κ=0.734) [45,46] over 892 double-annotated tweets. The test data sets were randomly selected from the corpora, and therefore, they can be considered a sample of the collected data. For Corpus 1, the test data contained 1000 tweets, among which political discussion was the dominant class (603/1000, 60.30%), followed by news (199/1000, 19.90%), whereas consumer feedback comprised 4.60% (46/1000) of the tweets. In contrast, consumer feedback comprised 57.90% (579/1000) of the tweets in Corpus 2, and 30.80% (308/1000) of the tweets could not be categorized, most of which were part of conversations and could not be understood without full context.

### Classification Results

The F_1_ scores for each class and the accuracies of the classifiers on the validation set and the test sets are presented in Table 2, including CIs estimated using bootstrapping, whereas the precisions and recalls are given in Table S6 of Multimedia Appendix 1. For the validation and test sets from Corpus 1, the classifiers showed high performance for political discussion, but relatively low performance for consumer feedback. This was expected based on the large imbalance described earlier. Among all the traditional classifiers tested, SVM performed the best on the validation set, with an F_1_ score of 0.53 on the consumer feedback. However, the F_1_ score on the consumer feedback on the test set from Corpus 1 was only 0.38. In contrast, we found that the BERT classifier had the highest F_1_ scores on consumer feedback for both the validation set (0.61) and the test set from Corpus 1 (0.58).

For the test set from Corpus 2, most of the classifiers performed well on the consumer feedback. Among the traditional classifiers, RF performed the best, with an F_1_ score of 0.82 on consumer feedback. On the other hand, BERT still performed the best, with a consumer feedback F_1_ score of 0.90.

As the BERT classifier performed the best in terms of accuracy and the consumer feedback F_1_ score on the validation set and the 2 test sets, we used the BERT classification for postclassification analysis.

### Error Analysis

We conducted a brief analysis of the errors made by the BERT-based classifier. We first calculated the confusion matrix for both test sets (Table 3). In Table 4, we provide examples of the most frequent classification errors, omitting unnecessary details. For Corpus 1, we highlighted that the classifier frequently misclassified political tweets as news or consumer feedback, and vice versa. This is not surprising because users sometimes commented on and discussed politics with personal experience, and some news content was related to opinions about the policy. We also highlighted that the uncategorized tweets, whose content is often not directly related to Medicaid or lack of information, are frequently misclassified as consumer feedback or political. The confusion between consumer feedback and political or uncategorized tweets, along with the low volume of consumer feedback, contributes to the low performance of consumer feedback. We also observed that some news tweets were confused with the information tweets because information is frequently spreading as news or blog articles.

**Table 3:** The BERT classifier’s confusion matrix on the test set

Data set and true value		Predicted value					
		Academic	Consumer	Information	News	Political	Other
**Test set (Corpus 1)**							
	Academic	13	0	1	4	2	0
	Consumer	0	26	0	0	18	2
	Information	0	0	27	9	10	3
	News	1	0	9	169	17	3
	Political	2	5	3	39	549	5
	Other	0	12	4	4	30	33
**Test set (Corpus 2)**							
	Academic	3	0	0	1	2	0
	Consumer	1	512	1	2	26	37
	Information	1	5	33	15	1	25
	News	0	0	5	11	3	2
	Political	0	0	0	1	5	0
	Other	1	36	15	7	6	243

**Table 4:** Examples of misclassified tweets by the BERT classifier on Corpus 1 and Corpus 2.

Data set, Tweets		True class (prediction)	Comments
**Test set (Corpus 1)**			
	I need this government shutdown to end because no one is going to call me to set up my Medicaid while it’s shutdown	Political (consumer)	Discussion about politics with personal experience
	“This is just cruelty and exclusion”: Amid Trump’s attack on poor, one million fewer kids receiving Medicaid and CHIP–Raw Story <URL>	Political (news)	Opinion on Medicaid policy presented as a news title
	<USERNAME> So do I! But I totally understand why some people really hate it. And yes... lack of Medicaid providers is a problem everywhere (I do accept it, but only have a mobile practice). Maybe contact your local health department and ask!	Consumer (political)	Customer’s discussion about Medicaid services. It may have been misclassified because of similarity to political discussion regarding Medicaid
	Thanks to <USERNAME> for this story about the bill ... Ohio leaving some military families with special needs children waiting for answers <URL>	News (political)	News about Medicaid policy reformation bill
	States that been successful in lowering substance use disorder rates have increased access to medicaid &amp; private insurance, and to MAT and naloxone. Thank you, NYT Editorial Board @NYTOpinion. <URL>	News (information)	News about information related to Medicaid
	3 Ways to increase Missouri Medicaid EMOMED Reimbursement <URL>	Information (news)	Information for Medicaid beneficiaries, presented as a blog article
	The Medicaid office I'm going to tomorrow opens at 7:30 am. I won't be there that early, but ugh.	Other (consumer)	Uncategorized because it is not about experience or question, but content indicates the user to be a customer
	<USERNAME> I hope someone will ask him “What's the difference between Medicaid and Medicare?”	Other (political)	Uncategorized because of lack of related content but is similar to political discussion
**Test set (Corpus 2)**			
	<organization_name> poorly worded	Consumer (other)	Most likely comment on customer service but hard to pick up by algorithm
	<organization_name> My pleasure!	Other (consumer)	Classified as others because of lack of information, but the algorithm might recognize that it could be a conversation between a customer and a customer representative

For Corpus 2, the dominant classes were consumer feedback and uncategorized tweets, and they were most frequently misclassified as each other. We suspect they were misclassified because tweets sometimes lacked context, making their meanings ambiguous and difficult for the machine to understand. For example, the tweet “<organization_name> poorly worded,” though ambiguous, might be understood as that some document for the customer or the customer service representative’s expression was poorly worded, and thus, we categorized it as consumer feedback. However, the machine learning algorithms were not capable of deciphering such implicit contexts: “poorly worded” is usually associated with a feedback and, in tweets directed to the agency’s handle, it is likely related to customer service. Similarly, the tweet “<organization_name> My pleasure!,” may belong to a conversation between a customer and a representative, but the lack of information renders it to the other class. However, machine learning could not capture this understanding.

### Postclassification Analyses: Data Distribution

**Figure 3:**
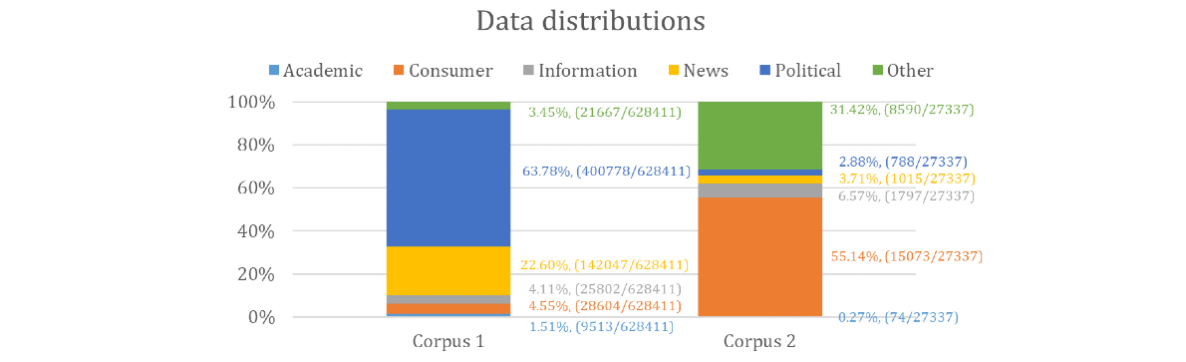
Postclassification class distributions among 2 corpora, as per the automatically classified tweets.

We applied the best-performing classifier (BERT) to label both corpora. The obtained class distributions are shown in Figure 3. We found that the majority of tweets in Corpus 1 were news (142047/628411, 22.60%) and political discussion (400778/628411, 63.78%), whereas consumer feedback accounted for only 4.55% (28604/628411), consistent with the data distribution of the test set of Corpus 1. The data distribution indicates that this corpus is suitable for analyzing chatter regarding political discussion or news. For Corpus 2, the majority of the tweets were labeled as consumer feedback (15073/27337, 55.14%) and uncategorized (8590/27337, 31.42%), which is also consistent with the data distribution in the test set.

### Postclassification Analyses: Content of Each Class in Corpus 1

We now briefly summarize the findings from content analyses of the tweets in Corpus 1 that contain the terms associated with “medicaid” to understand, from a high-level perspective, the contents within each category. The 10 highest ranking bigrams and trigrams detected by the TFIDF method are listed in Table S7 of Multimedia Appendix 1 [43]. Not surprisingly, the academic tweets are dominated by terms starting with “study...” and terms indicating research finding. Similarly, the information tweets contain terms related to “service,” “care,”... etc, consistent with information outreach. For the news tweets, we found that many tweets were about news on medicaid work requirements in Kentucky and Arkansas (blocked by the federal judge on March 27, 2019). In addition, “social security” and “Trump...” are also highly ranked among the news and political classes. For the tweets belonging to the consumer feedback, some of the high-ranking terms were shared with other classes (eg, “... insurance,” “social security,” or “... care”), whereas some were specific to this class (“make much” or “doesn cover”) and potentially indicated comments about Medicaid income cap and coverage.

We did not know the compositions of the 2 data sets that we had collected a priori. Thus, the results of our classification experiments provided us with very important knowledge about which type of Twitter data to use when conducting targeted studies about Medicaid or health services in general. For example, when studying consumer feedback, it is best to use data from Corpus 2 (ie, tweets containing Twitter handles of the MA or MCO); for studying public perceptions of political decisions, Corpus 1 would be more useful. Detailed content analyses of the tweets in each category, such as their temporal and geolocation-specific distributions, are likely to reveal more relevant information. However, such analyses are outside the scope of this study, and we plan to build on the NLP system described in this paper to conduct more thorough content analyses in the future.

### Postclassification Analyses: LDA Topic Modeling on Consumer Feedback

We found that the model with 20 topics achieved the highest coherence score. The top 20 words in each topic are listed in Multimedia Appendix 1, Table S8. We now summarize the main findings based on these top words, with example top words provided in parentheses. We deduced that this chatter contains discussion related to (1) applying for Medicaid, either for oneself or even family members (eg, *deny*, *apply*, *family*, and *child*); (2) Medicaid coverage for dentists, specialists, prescription medications, emergency department visits (eg, *cover*, *dentist*, *therapist*, *prescription*, *medication*, and *emergency*); (3) interacting with customer representatives, especially through phone (eg, *call*, *phone*, *hour*, *hold*, *tell*, and *wait*); (4) hospital-related bills (eg, *hospital*, *bill*, and *copay*); and (5) comparing different insurance plans (*switch*, *insurance*, *private*, and *plan*). The list of topics can be a guide to further categorize the consumer feedback chatter, which could lead to a more detailed analysis and even provide recommendations on how to further improve the Medicaid program. A more in-depth analysis is left for future work.

## Discussion

### Principal Findings

As many classification errors occur because the tweets lie in the boundary between 2 classes, we note that a multilabel classification scheme might improve the performance [47]. However, in the experiments conducted earlier in this project, we found that the multilabel scheme only improved the classification performance by a small margin, while making the annotation process more difficult. Thus, we focused on the single-label classification scheme in this work, leaving the development of multilabel models to future work.

In addition to multilabel classification models, the classification error might also be remedied by creating new categories for tweets lying at the boundary of current categories. For example, we can further divide the political discussion into 2 categories: discussion of policy without personal experiences or experiences from friends or relatives and discussion of policy with experiences as supporting evidence. The classification performance may be further improved by including more user profile information. For example, we can include features such as whether the account belongs to a news agency or if the user is affiliated with an academic organization or think tanks, which could improve the classification performance on the news class or the academic class. As the 2 corpora have very different distributions, developing a corpus-specific classifier might further improve the performance.

Although our content analysis is limited to the high-ranking TFIDF terms and LDA topic modeling on consumer feedback, additional analyses could include topic modeling of other chatter [44] or sentiment analysis to understand sentiments toward Medicaid in general or to specific aspects of the Medicaid program [8]. The manual analysis of selected samples can deepen the understanding of these topics and potentially generate recommendations toward policy change. We also note that content analysis can not only help researchers further understand the Medicaid chatter but it can also improve the classification performance in reverse.

### Limitations

This analysis has limitations related to the quality of Twitter data, which contain high volumes of noise that may affect the accuracy and generalizability of our content analyses and annotation guidelines. In addition, Twitter users may not be representative of Medicaid enrollees. Older age groups tend to be underrepresented among Twitter users [48], and more vulnerable populations who rely on Medicaid may not use this platform to discuss their health coverage.

### Conclusions

We have developed a social media mining system, involving NLP and machine learning, for continuously collecting and categorizing Twitter chatter about the Medicaid program. Our study demonstrates that it is possible to collect data about large, complex health services and coverage programs such as Medicaid using Twitter to obtain near real-time knowledge about consumer perceptions and opinions. The automatic classification of streaming data is crucial, specifically for smaller classes, such as consumer feedback, for studying targeted topics.

Our analysis can inform public health researchers on how to use public discussions about health programs and services, such as Medicaid. Similarly, our system can be deployed by research groups or Medicaid agencies for continuous, ongoing research on the evolution of public opinions on social media (eg, the impact of certain policy changes or rulings). We also note that although this work focuses on Medicaid, our methods and open source code can readily be applied to other health services. Annotated data and methods are available for future studies [49].

## Appendix

Multimedia Appendix 1 Supplementary materials, including tables and additional information.

## Abbreviations

BERT: bidirectional encoder representations from transformers
BLSTM: bidirectional long short-term memory
KNN: k-nearest neighbor
LDA: latent Dirichlet allocation
MA: Medicaid agency
MCO: Managed Care Organization
NB: naïve Bayes
NLP: natural language processing
NN: shallow neural network
RF: random forest
SVM: support vector machine
TFIDF: term frequency-inverse document frequency

## References

[1] Chen PS, Wu S, Yoon J (2004). The impact of online recommendations and consumer feedback on sales. Proceedings of the International Conference on Information Systems, ICIS 2004.

[2] Mudambi SM, Schuff D (2010). Research note: what makes a helpful online review? A study of customer reviews on Amazon.com. MIS Q.

[3] Hu M, Liu B (2004). Mining and summarizing customer reviews. Proceedings of the tenth ACM SIGKDD international conference on Knowledge discovery and data mining.

[4] Akay A, Dragomir A, Erlandsson B (2015). A Novel Data-Mining Approach Leveraging Social Media to Monitor Consumer Opinion of Sitagliptin. IEEE J. Biomed. Health Inform.

[5] Lim Y, Van Der Heide B (2014). Evaluating the wisdom of strangers: the perceived credibility of online consumer reviews on yelp. J Comput-Mediat Comm.

[6] Luca M (2016). Reviews, reputation, and revenue: the case of Yelp.com. Harvard Business School NOM Unit Working Paper.

[7] Yue L, Chen W, Li X, Zuo W, Yin M (2018). A survey of sentiment analysis in social media. Knowl Inf Syst.

[8] Liu B (2012). Sentiment analysis and opinion mining. Synthesis Lectures on Human Language Technologies.

[9] Broniatowski DA, Paul MJ, Dredze M (2013). National and local influenza surveillance through Twitter: an analysis of the 2012-2013 influenza epidemic. PLoS One.

[10] Sarker A, O'Connor Karen, Ginn R, Scotch M, Smith K, Malone D, Gonzalez G (2016). Social Media Mining for Toxicovigilance: Automatic Monitoring of Prescription Medication Abuse from Twitter. Drug Saf.

[11] O'Connor K, Pimpalkhute P, Nikfarjam A, Ginn R, Smith K, Gonzalez G (2014). Pharmacovigilance on Twitter? Mining tweets for adverse drug reactions. AMIA Annu Symp Proc.

[12] Griffis HM, Kilaru AS, Werner RM, Asch DA, Hershey JC, Hill S, Ha YP, Sellers A, Mahoney K, Merchant RM (2014). Use of social media across US hospitals: descriptive analysis of adoption and utilization. J Med Internet Res.

[13] Harris JK, Mueller NL, Snider D (2013). Social media adoption in local health departments nationwide. Am J Public Health.

[14] Glover M, Khalilzadeh O, Choy G, Prabhakar AM, Pandharipande PV, Gazelle GS (2015). Hospital Evaluations by Social Media: A Comparative Analysis of Facebook Ratings among Performance Outliers. J Gen Intern Med.

[15] Campbell L, Li Y (2018). Are Facebook user ratings associated with hospital cost, quality and patient satisfaction? A cross-sectional analysis of hospitals in New York State. BMJ Qual Saf.

[16] Hefele JG, Li Y, Campbell L, Barooah A, Wang J (2018). Nursing home Facebook reviews: who has them, and how do they relate to other measures of quality and experience?. BMJ Qual Saf.

[17] Hawkins JB, Brownstein JS, Tuli G, Runels T, Broecker K, Nsoesie EO, McIver DJ, Rozenblum R, Wright A, Bourgeois FT, Greaves F (2016). Measuring patient-perceived quality of care in US hospitals using Twitter. BMJ Qual Saf.

[18] Rastegar-Mojarad M, Ye Z, Wall D, Murali N, Lin S (2015). Collecting and Analyzing Patient Experiences of Health Care From Social Media. JMIR Res Protoc.

[19] Ranard BL, Werner RM, Antanavicius T, Schwartz HA, Smith RJ, Meisel ZF, Asch DA, Ungar LH, Merchant RM (2016). Yelp Reviews Of Hospital Care Can Supplement And Inform Traditional Surveys Of The Patient Experience Of Care. Health Aff (Millwood).

[20] van den Broek-Altenburg EM, Atherly AJ (2019). Using Social Media to Identify Consumers’ Sentiments towards Attributes of Health Insurance during Enrollment Season. Applied Sciences.

[21] Hatchard JL, Neto JQ, Vasilakis C, Evans-Reeves KA (2019). Tweeting about public health policy: social media response to the UK Government's announcement of a parliamentary vote on draft standardised packaging regulations. PLoS One.

[22] Medicaid enrollment changes following the ACA. Medicaid and CHIP Payment and Access Commission.

[23] Han B, Cook P, Baldwin T (2013). Lexical normalization for social media text. ACM Trans Intell Syst Technol.

[24] Sarker A, Gonzalez-Hernandez G (2018). An unsupervised and customizable misspelling generator for mining noisy health-related text sources. J Biomed Inform.

[25] Zhu JM, Sarker A, Gollust S, Merchant R, Grande D (2020). Characteristics of Twitter use by state medicaid programs in the United States: machine learning approach. J Med Internet Res.

[26] Martin PY, Turner BA (1986). Grounded theory and organizational research. J Appl Behav Sci.

[27] Rish I (2001). An empirical study of the naive Bayes classifier. Proceedings of IJCAI-01 workshop on Empirical Methods in AI.

[28] Cover TM, Hart PE (1967). Nearest neighbor pattern classification. IEEE Trans. Inform. Theory.

[29] Chang CC, Lin CJ (2011). LIBSVM: a library for support vector machines. ACM Trans Intell Syst Technol.

[30] Platt JC (1999). Probabilistic outputs for support vector machines and comparisons to regularized likelihood methods. Advances in large margin classifiers.

[31] Ho TK (1995). Random decision forests. Proceedings of 3rd International Conference on Document Analysis and Recognition.

[32] Hochreiter S, Schmidhuber J (1997). Long short-term memory. Neural Comput.

[33] Schuster M, Paliwal KK (1997). Bidirectional recurrent neural networks. IEEE Trans Signal Process.

[34] Devlin J, Chang MW, Lee K, Toutanova K (2018). Bert: pre-training of deep bidirectional transformers for language understanding. Computation and Language.

[35] Liu Y, Ott M, Goyal N, Du J, Joshi M, Chen D, Levy O, Lewis M, Zettlemoyer L, Stoyanov V (2019). Roberta: a robustly optimized bert pretraining approach. Computation and Language.

[36] Sarker A, Gonzalez G (2015). Portable automatic text classification for adverse drug reaction detection via multi-corpus training. J Biomed Inform.

[37] Pedregosa F, Varoquaux G, Gramfort A, Michel V, Thirion B, Grisel O, Blondel M, Prettenhofer P, Weiss R, Dubourg V, Vanderplas J, Passos A, Cournapeau D, Brucher M, Perrot M, Duchesnay E (2011). Scikit-learn: machine learning in python. J Mach Learn Res.

[38] (2015). Keras Computer Program.

[39] Wolf T, Debut L, Sanh V (2020). Transformers: State-of-the-Art Natural Language Processing. Proceedings of the 2020 Conference on Empirical Methods in Natural Language Processing: System Demonstrations.

[40] Nikfarjam A, Sarker A, O'Connor K, Ginn R, Gonzalez G (2015). Pharmacovigilance from social media: mining adverse drug reaction mentions using sequence labeling with word embedding cluster features. J Am Med Inform Assoc.

[41] Owoputi O, O’Connor B, Dyer C, Gimpel K, Schneider N (2012). Part-of-speech tagging for Twitter: Word clusters and other advances. Technical Report CMU-ML-12-107, Carnegie Mellon University.

[42] Pennington J, Socher R, Manning C (2014). Glove: Global vectors for word representation. Proceedings of the 2014 Conference on Empirical Methods in Natural Language Processing (EMNLP).

[43] Beel J, Gipp B, Langer S, Breitinger C (2015). Research-paper recommender systems: a literature survey. Int J Digit Libr.

[44] Blei DM, Ng AY, Jordan MI (2003). Latent Dirichlet Allocation. J Mach Learn Res.

[45] Viera AJ, Garrett JM (2005). Understanding interobserver agreement: the kappa statistic. Fam Med.

[46] Cohen J (1968). Weighted kappa: nominal scale agreement with provision for scaled disagreement or partial credit. Psychol Bull.

[47] Tsoumakas G, Katakis I (2007). Multi-label classification: an overview. Int J Data Warehous Mining (IJDWM).

[48] Wojcik S, Hughes A (2019). Sizing up Twitter users. Pew Research Center.

[49] Bitbucket.

